# From economic difficulties to psychological maladjustment in Italian women during the Covid-19 pandemic: does marital dissatisfaction moderate or mediate this association?

**DOI:** 10.3389/fpsyg.2023.1166049

**Published:** 2023-06-22

**Authors:** Elena Camisasca, Venusia Covelli, Dario Cafagna, Gian Mauro Manzoni, Manuela Cantoia, Alessandra Bavagnoli, Pietro Crescenzo, Vincenzo Marsicovetere, Mario Pesce, Marina Angela Visco

**Affiliations:** ^1^Faculty of Psychology, eCampus University, Novedrate, Como, Italy; ^2^Department of Education, Psychology and Communication, University of Bari Aldo Moro, Bari, Italy

**Keywords:** COVID-19, economic difficulties, perceived stress, marital satisfaction, psychological maladjustment, women

## Abstract

**Introduction:**

The empirical study about the negative impact of economic difficulties due to Covid- 19 on the psychological well-being of Italian women by considering perceived stress and marital satisfaction is an area worthy of investigation. The study explored these variables by hypothesizing that marital satisfaction (DAS) could moderate or mediate the links between economic difficulties, perceived stress (PSS), and psychological maladjustment (PGWBI).

**Methods:**

A total of 320 Italian women completed an online survey about the study’s variables during the lockdown period. Women’s perceptions of economic difficulties due to COVID- 19 restrictions were detected through an ad-hoc specific question. Perceived stress, marital satisfaction and psychological maladjustment were assessed by standardized questionnaires (Perceived Stress Scale 10, Dyadic Satisfaction Scale and Psychological General Well-being Inventory).

**Results:**

39.7% of women who answered the online survey said that the Covid-19 significantly impacted their family income. Results indicated that marital satisfaction did not moderate the associations investigated. Conversely, data showed how economic difficulties (X) predicted lower psychological maladjustment through the mediation of perceived Stress (M1), which, in turn, was associated with higher levels of marital dissatisfaction (M2).

**Conclusion:**

The results of the present study confirm the significant role of marital dissatisfaction in explaining the indirect effects of economic difficulties on psychological maladjustment in women. In particular, they indicated a significant spillover effect which transmitted strains experienced in one domain (economic difficulties) to another (the dissatisfaction of the couple), which in turn affected the psychological maladjustment.

## Introduction

The lockdowns imposed because of the COVID-19 pandemic in European Countries and overseas impacted people at different levels, with consequences on the psychological well-being of the people involved as well as implications for their income (Brooks et al., 2020; International Labour Organization, 2020). Confining lockdown measures, including self-isolation, and the suspension of superfluous activities, led to a consequent reduction in work hours, income loss, and job loss for many workers (Crayne, 2020; Hensher, 2020; Matthews et al., 2021; McDowell et al., 2021). The economic difficulties and the worries and uncertainty of the future led to a condition of financial strain - the subjective stress about financial concerns – which impacted many individuals and families (Friedline et al., 2021; Hertz-Palmor et al., 2021). Several surveys undertaken during the COVID-19 pandemic showed that unemployment, economic difficulties, and financial strain predicted higher levels of psychological distress and mental health disorders (Achdut and Refaeli, 2020; Kelley et al., 2020; Armour et al., 2021). More specifically, both loss of job/income (objective financial hardship) and perceptions of financial strain (the subjective feeling of economic well-being) were related to psychological maladjustment in terms of anxiety and depressive symptoms (Hertz-Palmor et al., 2021). Some authors (Ruengorn et al., 2021; Trógolo et al., 2022) suggested that the subjective perceptions of financial strain had more damaging effects on mental health (particularly on anxiety, depression, and perceived stress) than objective economic indicators (i.e., income or job loss). Conversely, other scholars suggested that objective financial hardship, such as job loss, unemployment, and lower income or loss of income, during the pandemic led to more psychological distress and depressive symptoms (Crayne, 2020; Pierce et al., 2020; van der Velden et al., 2020). Specifically, some European studies (Pierce et al., 2020; van der Velden et al., 2020) showed that, during the pandemic, people who lost their job, were unemployed, or had no income were more distressed than individuals who were employed or already unemployed before COVID-19. Similarly, other studies from the U.S. demonstrated that low income, lack of savings, and unemployment were risk factors for depression during the COVID-19 pandemic compared with pre-pandemic (Ettman et al., 2020; Fitzpatrick et al., 2020; Twenge and Joiner, 2020).

These findings outlined the associations between the COVID-19 related decrease in financial security and the deterioration in mental health. Indeed, individuals with higher economic difficulties and financial strain could perceive more psychological distress, which in turn promoted the onset of anxiety, depression, and a series of other mental health problems (Wilson et al., 2020; Witteveen and Velthorst, 2020; Hertz-Palmor et al., 2021). Specifically, some studies demonstrated that psychological distress due to financial strain led to the onset of anxious and depressive feelings or the exacerbation of depression (Wilson et al., 2020; Witteveen and Velthorst, 2020; Hertz-Palmor et al., 2021).

During the pandemic, economic difficulties and financial strain also harmed couple relationships by promoting greater dissatisfaction with marriage and lower relationship commitment, resulting in more frequent conflicts (see Epifani et al., 2021 for a review). For example, Balzarini et al. (2020) showed how economic difficulties, perceived stress, and confining lockdown measures were negatively associated with the couple’s relationship quality. In a recent meta-analysis, Falconier and Jackson (2020) indicated how economic strain impacted the quality of the couple’s relationship functioning, independently from sociodemographic factors, study design, or sample type.

The impact of financial stress on the quality of the couple’s relationships and psychological well-being has already been pointed out in the past literature (Kelley et al., 2022). Several studies showed how objective financial stressors, such as unstable income and perceived financial stress, predicted lower levels of marital satisfaction (Gudmunson et al., 2007; Archuleta et al., 2011; Stewart et al., 2017) and an increase in marital conflicts and marital dissolution (Dew et al., 2012). To explain the association between external stress (such as economic difficulties) and marital satisfaction, literature introduced the notion of “spillover effect.” The spillover effect refers to a situation when stress felt in one domain (e.g., work or couple) is transmitted to another domain (e.g., family/parenting behaviors) and negatively impacts the well-being of individuals in their other roles (Kouros et al., 2014). Many studies (e.g., Frone, 2003; Ilies et al., 2007; Kouros et al., 2014) have found that job stressors can spill over to the family domain and increase partner distress and marital dissatisfaction. More specifically, according to Pietromonaco and Overall (2020), unemployment, economic strain, and work difficulties could spill over and negatively impact the quality of the couples’ relationship by creating a context in which partners are distracted, fatigued, or overwhelmed. As a result, the partners could become conflictual and critical, give less support, and become less happy with their romantic relationship.

Moreover, literature indicated how deteriorated marital relationships could be a link between economic difficulties and psychological maladjustment. For example, Romeo et al. (2022) suggested that people who indicated a negative impact of COVID-19 on their family income reported a deteriorated couple relationships and higher levels of internalizing symptoms and post-traumatic symptoms.

During the Covid-19 pandemic, the study of Pieh et al. (2020, 2021) showed how the couple’s relationship quality was related to mental health and well-being. They found that good relationship quality predicted higher levels of psychological well-being and mental health.

The protective role of a good marital relationship on mental health was also outlined in several previous studies (Horwitz et al., 1996; Simon, 2002; Williams, 2003; Thoits, 2011) that showed how, according to the stress-buffering model (Cohen and Wills, 1985), significant others may buffer the harmful effects of stressful events on mental health through providing emotional support and active coping assistance. Indeed, if individuals receive emotional support from their significant others, in case of external stressors, they will be more effective in mobilizing their resources and become less prone to suffer from psychological maladjustment (Jesus et al., 2016; Viseu et al., 2018).

With specific reference to the negative impact of economic difficulties on mental health, some researchers (Conger et al., 1999; Lincoln and Chae, 2010) indicated that a good marital relationship might protect against economic hardship and reduce some negative affect that frequently accompanies economic pressure. More specifically, Lincoln and Chae (2010) investigated whether marital satisfaction moderates the influence of economic difficulties on psychological distress among African Americans. Results showed how marital satisfaction had a protective role in moderating the negative effects of financial difficulties on psychological distress. Moreover, Viseu et al. (2018) assessed the association between economic difficulties and the symptoms of stress, anxiety, and depression by testing the moderating effect of informational, practical, and emotional support from significant others on these associations. Results showed the moderating effects of support from significant others regarding the association between economic stress and mental health. Similarly, another recent study (Chai, 2023) investigated the association between economic difficulties and psychological distress by exploring how this link could be mediated by sleep problems and moderated by marital status (a moderated mediation model). Results showed that economic problems predicted psychological distress through the partial mediation of sleep problems. Moreover, marital status moderated the associations between economic problems and psychological distress and between sleep difficulties and psychological distress.

Considering the above-cited literature, we were interested in exploring a line of research that is almost blank and rife with potential for exploration. Specifically, we decided to investigate the predictive role of economic difficulties on psychological maladjustment in Italian women by focusing on marital satisfaction as both a potential protective factor (moderator) or explicative mechanism of this association (mediator).

The present study had two aims. The first one is to explore the role of marital satisfaction in moderating the indirect effects of economic difficulties on women’s psychological maladjustment through the mediation of perceived stress. According to the literature (Lincoln and Chae, 2010; Chai, 2023), we supposed that marital satisfaction could exert a protective role by reducing the negative impact of economic difficulties and perceived stress on women’s psychological adjustment.

The second aim is to explore the potential spillover effect from economic difficulties to women’s psychological maladjustment by considering the serial mediation of perceived stress and marital dissatisfaction. In other words, based on the literature (Epifani et al., 2021; Friedline et al., 2021; Hertz-Palmor et al., 2021), we hypothesized that economic difficulties during the Covid-19 outbreak could predict the onset of higher levels of perceived stress which, in turn, negatively affects the couple’s relationship with consequences for psychological adjustment.

The focus is on women because research (Reizer et al., 2020) has shown that they were more prone than men to stress and psychological adjustment problems during the COVID-19 period. Specifically, women reported higher levels of internalizing symptoms, depression, anxiety, and stress during the COVID-19 pandemic in China (Wang et al., 2020) and in 25 European countries (Gamonal-Limcaoco et al., 2020).

## Methods

### Procedure

A web-based survey was created on the Qualtrics Platform and spread through different social media sites (the survey link was posted on Facebook, LinkedIn, and Instagram, with a snowball sampling via WhatsApp) from April 2020 to July 2020. The survey was directed to married or cohabiting women, who were asked to answer some questions about sociodemographic characteristics and to fill out some questionnaires about the study’s variables during the lockdown period.

Participation in the survey was voluntary, and women had to give their informed consent to participate as well as to data treatment before starting to answer the questions. The survey took approximately 20 min to be completed. In the treatment of the participants, we have followed APA guidelines and the 1964 Helsinki Declaration and its later amendments or comparable ethical standards.

### Participants

For the survey (which addressed married or cohabitant women with children), 320 Italian women aged between 30 and 58 years old (*M* = 42.48; *DS* = 6.0) agreed to participate and provided data. A summary of the participant’s demographic characteristics is displayed in Table 1. In short, a significant number of women were married (76%; duration of marriage or cohabitation: *M* = 13.0; *DS* = 7.8), well educated (48% with more than a high school degree), and with a total family income ranging from 17.000 to 35.000 euro (49%).

**Table 1:** Demographic characteristics of the study sample (*N* = 320).

		*N*	*Percentage*
Education	High School Degree or Less	166	52.0%
	More than High Scool Degree	154	48.0%
Profession pre Covid-19	Simple professions/Housewife/Unemployed/	30	9.4%
	Skilled Workers	33	10.3%
	Service and sales occupations	*131*	40.9%
	Professionals in scientific, tecnical and human fields	*32*	10%
	Lagislators, Mangers and Executives	*94*	29.4%
Professional Condition in the Lockdown Period	Housewife/Unemployed	30	9.4%
	Face to Face Working	50	15.6%
	Smart Working	148	46.3%
	Job Interruption	88	27.5%
	Job Loss	8	2.5%
Relational Status	Married	243	75.9%
	Cohabitants	77	24.1%
Family Income	17.000 Euro or less	147	45.9%
	From 17.000 to 35.000 Euro	156	48.8%
	More than 35.000 Euro	17	5.3%

### Measures

#### Economic difficulties

Women’s perceptions of Economic Difficulties due to COVID-19 restrictions were detected through an *ad-hoc* specific question: “*Did the restrictive measures due to the Covid-19 emergency negatively impact your family income, with a consequent condition of concern, tension, and stress?.”* The answers were dichotomously coded as “Yes” =1; or “Not” = 0. We used a dichotomous response as we are primarily interested in detecting the appraisal of the presence/absence of a negative economic impact of Covid-19. 39.7% of the sample indicated that the restrictive measures negatively impacted their family income.

#### Perceived stress

The women’s perceived stress was assessed with the Perceived Stress Scale (PSS-10). The PSS-10 (Cohen et al., 1983; 1988; Italian version Fossati, 2010) is a self-report measure consisting of 10 items aimed to measure self-reported stress in terms of “H*ow unpredictable, uncontrollable, and over-loaded respondents find their lives*.” Participants are asked to answer each question on a Likert scale ranging from 0 (never) to 4 (very often). The PSS-10 total score ranges from 0 to 40, with higher scores indicating higher levels of perceived stress. In this study, the value of the internal consistency of the measure was found to be high for the *Perceived Stress Scale* (α = 0.84).

#### Marital satisfaction

The women’s marital satisfaction was measured with the Dyadic Adjustment Scale (DAS; Spanier, 1976); Italian validation by Gentili et al. (2002). The DAS is a measure that assesses the quality of marital adjustment, and it has four subscales: (1) *Dyadic Consensus* (13 items), (2) *Dyadic Satisfaction* (10 items), (3) *Affective Expression* (4 items), and (4) *Dyadic Cohesion* (5 items). In the present study, due to the survey length, we administered only the *Dyadic Satisfaction* items to gain information about the level of women’s *marital satisfaction* (in terms of positive interactions or conflicts and thoughts of separation/divorce). In the study of the Italian validation of the DAS (Gentili et al., 2002), the internal consistency value for Marital *Satisfaction* was: α = 0.87, in this sample, the internal consistency of this Scale was also high (α = 0.81).

#### Psychological maladjustment

The participant’s psychological maladjustment was assessed using the Psychological General Well-being Inventory **(**PGWB; Dupuy, 1984). We used the Italian Short Version of the measure (Grossi et al., 2006), which is composed of six items that refer to these sub-scales: positive well-being, self-control, general health, vitality, anxiety, and depression. Higher scores show better psychological adjustment. In our sample, the internal consistency of the Total Score was high: α = 0.86.

### Data analysis

Point-serial correlations were used to investigate the associations between the variables.

Measures of central tendency (mean or median and absolute and relative frequencies) and dispersion (standard or interquartile deviation) were first computed to describe the sample. Descriptive statistics such as Skewness, Kurtosis, and boxplot were also used to inspect data distributions and to find outliers.

A Point-biserial correlation matrix was computed to investigate the associations between the predictor economic difficulties (a categorical variable) and the other variables of the study. Path analyses were performed to test the hypothesized relationships within a moderated mediation model and a serial mediation model. We used the Process Macro for SPSS (Hayes and Preacher, 2013) with Model 59 for the moderated mediation model and with Model 6 for the serial mediation model, which was estimated with a bias-corrected bootstrap sampling method (5,000 samples) because it is particularly suitable for small samples (Hayes, 2012). “A value of *p* of 0.05 was set as the critical level for statistical significance (for the analysis of indirect effects, if the 95% confidence interval includes 0, then the indirect effect is not significant at the 0.05 level, while if the interval does not include 0, is not in the interval then the indirect effect is statistically significant at the 0.05 level” (Hayes, 2013, p. 633).

The first model allowed exploring whether marital satisfaction (W) moderated the indirect effects of economic difficulties (X) on psychological maladjustment(Y) through perceived Stress (M).

The serial mediation model allowed for exploring the indirect effects of economic difficulties (X) on psychological maladjustment (Y) through perceived stress (M1) and marital satisfaction (M2) as serial mediators.

## Results

### Descriptive and correlational analyses

Descriptive statistics, point-biserial, and Pearson correlations are reported in Table 2. Means and standard deviations of scores in all scales were similar to those found in other Italian and international studies with normative samples (Grossi et al., 2006; Camisasca et al., 2016, 2019; Grabowski et al., 2021). Economic difficulties were significantly associated with perceived stress, marital satisfaction, and psychological maladjustment (*r* from −0.13 to 0.17), and perceived stress was correlated with both marital dissatisfaction (*r* = 0.27) and psychological maladjustment (*r* = −0.69).

**Table 2:** Descriptive, point-biserial, and Pearson correlational results.

	*M*	*SD*	*Percentage*	1	2	3	4
1. Economic difficulties (Specific question)	–	–	39.7%	–			
2. Perceived stress (PSS-10)	18.21	7.0	–	0.178^**^	–		
3. Marital satisfaction (DAS)	39.77	5.9	–	−0.131^*^	0.270^**^	–	
4. Psychological maladjustment (PGWBI-S)	17.50	5.1	–	−0.138^*^	−0.699^**^	0.276^**^	-

*N* = 320; M, means; SD, standard deviation; ^**^p < 0.01; ^*^p < 0.05.

### The moderated mediation model

**Figure 1:**
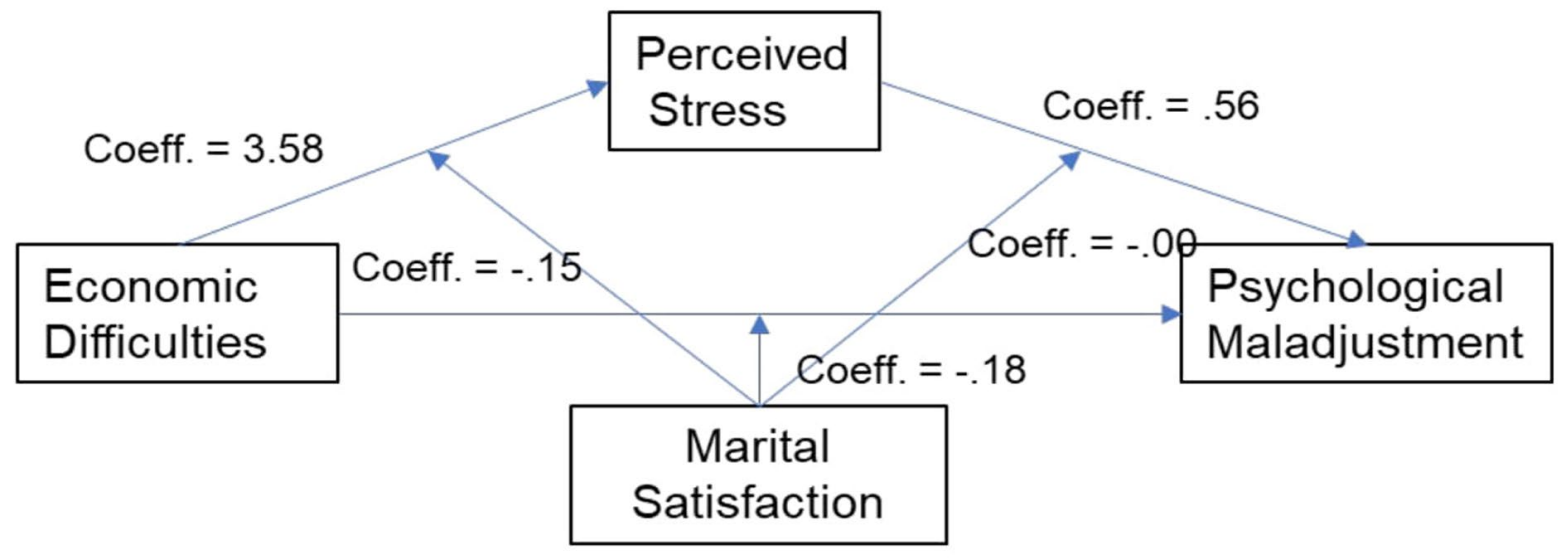
The moderated mediation model.

This model (see Figure 1) allowed exploring whether marital satisfaction (W) moderated the indirect effects of economic difficulties (X) on psychological maladjustment (Y) through perceived stress (M). In the analyses, we also considered the effect of family income as a possible covariate.

Results showed that economic difficulties resulted in predicting the perceived stress (Coeff. = 3.58; *p* < 0.01; LLCI-ULCI: 0.58;6.59), while marital satisfaction did not moderate this association (Coeff. = −0.15; *p* 0.16; LLCI-ULCI: −0.36;0.06). Marital satisfaction did not even moderate the effect of economic difficulties on the psychological maladjustment (Coeff. = 0.18; *p* 0.08; LLCI-ULCI: −0.40;0.02) nor the effects of perceived stress on psychological maladjustment (Coeff. =0.003; *p* 0.06; LLCI-ULCI: −0.001;0.01). Finally, the indirect effect of economic difficulties on women’s psychological maladjustment through the mediation of perceived stress (*R*^2^ = 0.50; *F* = 53.03; *p* < 0.01; Eff. =0.37; LLCI-ULCI: −1.29; −2.04), was not moderated by marital satisfaction (*Index of Moderated mediation;* −0.08; LLCI-ULCI: −0.20;0.09). The covariate family income did not exert a predictive role on both perceived stress (Coeff. = 0.67; *p* = 0.06; LLCI-ULCI: −0.04;1,40) and psychological maladjustment (Coeff. = −0.16; *p >* 0.05; LLCI-ULCI: −0.53;0.20).

### The serial mediation model

**Figure 2:**
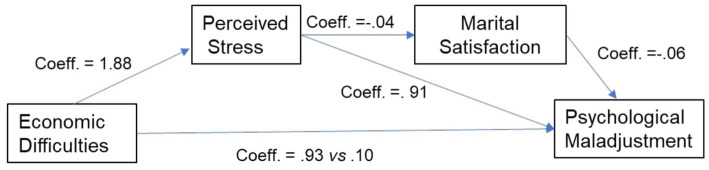
The serial mediation model.

This model (see Figure 2) allowed for exploring the indirect effects of economic difficulties (X) on psychological maladjustment (Y) through perceived stress (M1) and marital satisfaction (M2) as serial mediators. In the analyses, we also considered the effects of family income as a possible covariate.

The total effect of economic difficulties on psychological maladjustment was statistically significant (Coeff. =0.93, *p* < 0.05; *c path*), while the direct effect was not found to be significant (Coeff. = 0.10 *p* = 0.81; *c path*). Additionally, significant indirect effects were found (*R*^2^ = 0.49; *F* = 104.25; *p* < 0.001). In particular, the first indirect pathway passed from X (economic difficulties) to Y (psychological maladjustment) through M1 (perceived Stress: Coeff. = 0.91; LLCI-ULCI: −1.98−0.09). The second indirect pathway passed from X (economic difficulties) to Y (psychological maladjustment) through M1 (perceived stress) negatively affecting M2, marital satisfaction (Coeff. = −0.04; LLCI-ULCI: −0.10;.−0.00).

The covariate family income did not exert a predictive role on either perceived stress (Coeff. = 0.66; *p* = 0.06; LLCI-ULCI: −0.02;1.34) or psychological maladjustment (Coeff. = −0.18; *p >* 0.05; LLCI-ULCI: −0.54;0.18).

## Discussion

The Covid-19 pandemic involved an increase in economic difficulties with a consequent perceived psychological stress and a worsening of both mental health (Codagnone et al., 2020) and quality of family relationships (Pietromonaco and Overall, 2022). Thus, during the Covid-19 pandemic, some studies explored the predictive role of economic difficulties on both mental health and couple relationships (Balzarini et al., 2020; Falconier and Jackson, 2020). However, no study focused on marital satisfaction as a potential moderator or mediator of the effects of these associations. For this reason, we conducted a study in order to: (1) investigate the role of marital satisfaction in moderating the indirect effects of economic difficulties on psychological maladjustment through the mediation of perceived stress and (2) explore the potential spillover effect from economic difficulties to psychological maladjustment through the serial mediation of perceived stress and marital dissatisfaction.

Regarding the first aim, path analyses indicated that marital satisfaction did not moderate the indirect effects of economic difficulties on psychological maladjustment. In other words, we did not detect a protective role of marital satisfaction in decreasing the predictive impact of economic difficulties on psychological maladjustment. Results showed that economic difficulties predicted perceived stress which, in turn, predicted psychological maladjustment. However, these associations were not moderated by marital satisfaction.

The negative impact of economic difficulties on psychological maladjustment is consistent with the results of other contemporary studies, which showed that, aside from the utilitarian aspect of compensation, individuals derive significant meaning and value from their work (Crayne, 2020; Matthews et al., 2021; McDowell et al., 2021). As Crayne (2020, p. 180) suggested, “Work is a source of motivation and expression of personal beliefs that people hold as inextricable from their self-concept. Moreover, workplaces are a primary source of high-quality interpersonal interaction and relationship-building for many adults. Therefore, given the considerable proportion of adult life generally spent at work, it is reasonable that economic difficulties and work restrictions during the Covid-19 pandemic could have negatively contributed to psychological maladjustment.” However, in contrast with the buffering hypothesis (Cohen and Wills, 1985), which states that some factors, such as marital satisfaction (Rosand et al., 2011, 2012), may protect against severe effects of certain strains, this study’s results did not confirm the protective role of marital satisfaction. This finding aligns with the one reported by Balzarini et al. (2022, p. 24), who stated that “partner responsiveness is less effective in alleviating the strain from financial stress and financial concerns may be less mitigable through a partner’s support”. That is, when a partner feels lonely or stressed, a responsive partner could provide support through either spending time with his/her partner (to relieve loneliness) or being supportive of the stress. However, a partner’s support may not help relieve the financial strain because economic stressors may persist despite a partner’s responsiveness (e.g., if an individual loses a job, a partner’s support and responsiveness might not be useful to mitigate financial concerns and economic difficulties). In line with our results, Shi and Whisman (2023) in a recent longitudinal study showed that marital satisfaction did not moderate the association between acute stressful life events and depressive symptoms.

Concerning the second aim, results showed a significant *spillover effect* from economic difficulties to psychological maladjustment. The spillover effect was detected through higher levels of perceived stress and marital dissatisfaction. Our results about the predictive effect of economic difficulties on perceived stress are consistent with those of the literature (Harvey and Bray, 1991; Achdut and Refaeli, 2020; Friedline et al., 2021; Hertz-Palmor et al., 2021), which showed that unemployment and economic difficulties had high psychological costs, including the potential loss of meaning in life, impairment of personal identity, and the reduction of self-esteem that an individual typically draws from his/her job. Therefore, economic difficulties and strain promoted higher worry, tension, unhappiness, pessimism, and perceived stress.

Our data also indicated that the adverse emotional climate due to perceived stress spilled over to the couple’s relationship by increasing marital dissatisfaction and unhappiness. This result is also consistent with the literature (Pietromonaco and Overall, 2020; Işık and Kaya, 2022), which showed that increased levels of perceived stress led to decreased spousal support and marital satisfaction during the Covid-19 pandemic. Specifically, Pietromonaco and Overall (2020, p. 4) argued that “external stress made it difficult for partners to be responsive to each other because they were distracted, fatigued, or overwhelmed. As a result of this emotional climate, partners can become overly critical or argumentative, blame their partner, provide poorer support, and, over time, become less satisfied with their partner and relationship.” Again, Turliuc and Candel (2021) indicated that higher levels of external stress were associated with subsequent lower marital satisfaction during the Covid-19 pandemic, particularly for women.

Another significant result of our study is that the quality of the couple’s relationship, denoted by unhappiness and dissatisfaction, was directly associated with psychological maladjustment in women. This result is in line with previous studies (see Falconier et al., 2015), which showed that extra-dyadic stress is directly and indirectly related to lower psychological well-being through increased intra-dyadic stress from relationship problems. In particular, the female extra-dyadic and intra-dyadic stress negatively affected relationship satisfaction and posed more risks for marital quality.

In sum, the results of the present study confirm the significant role of marital dissatisfaction in explaining the indirect effects of economic difficulties on psychological maladjustment in women. In particular, they indicated a significant spillover effect which transmitted strains experienced in one domain (economic difficulties) to another (the dissatisfaction of the couple), which in turn affected the psychological maladjustment.

### Limitations and future directions of research

A series of limitations affect the findings of this study. The first one is that only one individual in the couple (a woman) was involved, preventing the analyses of the interplay between the couple’s members. Examining both partners and using dyadic data would have improved the knowledge of the associations of the considered variables. Second, the cross-section design of the study cannot demonstrate any causal effect. Results should thus be interpreted cautiously, and bi-directional effects could be considered. For example, we could also explore how economic difficulties affect psychological maladjustment, which in turn could be associated with marital dissatisfaction. Third, the sociodemographic homogeneity of participants and the small sample size limit the generalizability of results, even though the bootstrapping method could partly overcome this limitation. Another limitation is due to the single-item question used to assess the economic difficulties. We used a single-item query with a dichotomic answer to detect the presence/absence of economic difficulties (due to the pandemic). The use of an interval-level scale could have allowed a better and more detailed understanding of the degree of Covid-19 economic impact. Moreover, although we considered the family income as a covariate in the mediational analyses, the exploration, in further research, of other variables (the sample’s professional condition before and during the Covid pandemic) could be useful to better understand the associations investigated.

Moreover, another limitation consisted in administering only the items of the subscale dyadic satisfaction of the Dyadic Adjustment Scale (DAS). Indeed, exploring the other dimensions of this scale could allow an in-depth understanding of the role of the quality of the couple’s relationship in the associations investigated. A last limitation is related to the self-reported data because it is well known that self-report measures assessing sensitive information can be affected by social desirability, which could have inflated some associations.

Despite these limitations, the results of the present study can contribute to the advancement of knowledge about the effect that economic difficulties during the Covid-19 pandemic had on women’s psychological well-being through the mediation of both perceived stress and marital dissatisfaction. Longer-term studies could indicate whether these changes in relationship satisfaction remain a temporary phenomenon or will continue after the Covid-19 pandemic and, eventually, substantiate increased risks of partnership dissolution. Moreover, our results substantiate the importance of better understanding the associations between economic difficulties, marital satisfaction, and psychological maladjustment by assuming the interdependence between husband and wife. Therefore, future studies could consider the crucial aspects of interpersonal interaction in intimate relationships using an analysis method focused on the internal correlation of couple data. In this perspective, the actor–partner interdependence model (APIM, Kenny and Cook, 1999) can be used to investigate dyadic paired data, which can be examined directly at the dyadic level, as well as roles and partnerships.

## Data availability statement

The raw data supporting the conclusions of this article will be made available by the authors, without undue reservation.

## Ethics statement

Ethical review and approval was not required for the study on human participants in accordance with the local legislation and institutional requirements. The patients/participants provided their written informed consent to participate in this study.

## Author contributions

All authors listed have made a substantial, direct, and intellectual contribution to the work and approved it for publication.

## Conflict of interest

The authors declare that the research was conducted in the absence of any commercial or financial relationships that could be construed as a potential conflict of interest.

## Publisher’s note

All claims expressed in this article are solely those of the authors and do not necessarily represent those of their affiliated organizations, or those of the publisher, the editors and the reviewers. Any product that may be evaluated in this article, or claim that may be made by its manufacturer, is not guaranteed or endorsed by the publisher.
